# Limited role of regulatory T cells during acute Theiler virus-induced encephalitis in resistant C57BL/6 mice

**DOI:** 10.1186/s12974-014-0180-9

**Published:** 2014-11-13

**Authors:** Chittappen K Prajeeth, Andreas Beineke, Cut Dahlia Iskandar, Viktoria Gudi, Vanessa Herder, Ingo Gerhauser, Verena Haist, René Teich, Jochen Huehn, Wolfgang Baumgärtner, Martin Stangel

**Affiliations:** Department of Neurology, Clinical Neuroimmunology and Neurochemistry, Hannover Medical School, Carl-Neuberg-Str. 1, Hannover, 30625 Germany; Department of Pathology, University of Veterinary Medicine Hannover, Bünteweg 17, Hannover, D-30559 Germany; Experimental Immunology, Helmholtz Centre for Infection Research, Inhoffenstr. 7, Braunschweig, D-38124 Germany; Center of Systems Neuroscience, Hannover, Germany

**Keywords:** Regulatory T cells, Theiler’s virus, Interleukin-10

## Abstract

**Background:**

Theiler’s murine encephalomyelitis virus (TMEV) infection represents a commonly used infectious animal model to study various aspects of the pathogenesis of multiple sclerosis (MS). In susceptible SJL mice, dominant activity of Foxp3^+^ CD4^+^ regulatory T cells (Tregs) in the CNS partly contributes to viral persistence and progressive demyelination. On the other hand, resistant C57BL/6 mice rapidly clear the virus by mounting a strong antiviral immune response. However, very little is known about the role of Tregs in regulating antiviral responses during acute encephalitis in resistant mouse strains.

**Methods:**

In this study, we used DEREG mice that express the diphtheria toxin (DT) receptor under control of the *foxp3* locus to selectively deplete Foxp3^+^ Tregs by injection of DT prior to infection and studied the effect of Treg depletion on the course of acute Theiler’s murine encephalomyelitis (TME).

**Results:**

As expected, DEREG mice that are on a C57BL/6 background were resistant to TMEV infection and cleared the virus within days of infection, regardless of the presence or absence of Tregs. Nevertheless, in the absence of Tregs we observed priming of stronger effector T cell responses in the periphery, which subsequently resulted in a transient increase in the frequency of IFNγ-producing T cells in the brain at an early stage of infection. Histological and flow cytometric analysis revealed that this transiently increased frequency of brain-infiltrating IFNγ-producing T cells in Treg-depleted mice neither led to an augmented antiviral response nor enhanced inflammation-mediated tissue damage. Intriguingly, Treg depletion did not change the expression of IL-10 in the infected brain, which might play a role for dampening the inflammatory damage caused by the increased number of effector T cells.

**Conclusion:**

We therefore propose that unlike susceptible mice strains, interfering with the Treg compartment of resistant mice only has negligible effects on virus-induced pathologies in the CNS. Furthermore, in the absence of Tregs, local anti-inflammatory mechanisms might limit the extent of damage caused by strong anti-viral response in the CNS.

**Electronic supplementary material:**

The online version of this article (doi:10.1186/s12974-014-0180-9) contains supplementary material, which is available to authorized users.

## Background

Multiple sclerosis (MS), one of the most frequent central nervous system (CNS) diseases in young adults, is a chronic demyelinating disease of unknown etiology and probably multifactorial causes. Based on the generation of myelin-specific immune responses, MS is regarded as an autoimmune disease [1,2], possibly triggered by virus infections [3,4]. Due to clinical and pathological similarities, Theiler’s murine encephalomyelitis (TME) represents a commonly used infectious animal model for the chronic-progressive form of MS in humans [5]. Here, intracerebral infection of mice with the low virulent BeAn-strain of the Theiler’s murine encephalomyelitis virus (TMEV) causes an acute transient polioencephalitis [6], characterized by the infiltration of virus-specific lymphocytes [7]. Inadequate viral clearance in SJL mice leads to persistent infection of the CNS and immune-mediated spinal cord demyelination [6,8-10]. In contrast, resistant C57BL/6 mice eliminate the virus from the CNS by specific cellular immunity, including effective CD4^+^ and CD8^+^ T cell responses during the acute infection phase [11].

Regulatory T cells (Tregs), characterized by expression of the transcription factor forkhead box P3 (Foxp3), play a key role in the maintenance of immunological tolerance and prevent immunopathology [12-17]. However, in viral diseases Tregs can exhibit both beneficial effects by reducing immune-mediated tissue damage and detrimental effects due to their immunosuppressive properties, causing disease exacerbation or viral persistence, respectively. Recently, rapid expansion of Tregs associated with an increased expression of the immunosuppressive cytokine IL-10 has been demonstrated in the brain of susceptible SJL mice but not in resistant C57BL/6 mice following TMEV infection [11,18]. Moreover, functional inactivation of Tregs by anti-CD25 antibodies prior to infection results in an enhanced virus-specific immunity, reduced viral load, and delayed disease progression, while the adoptive transfer of Tregs leads to disease exacerbation in TMEV-infected SJL mice [11,19]. However, these treatments have failed to influence the disease course in resistant mice strains (C57BL/6), demonstrating the complexity of protective immune responses in infectious CNS disorders.

In order to obtain further insights into the relevance of Tregs for disease resistance and antiviral immunity in TME, the kinetics of CNS-infiltrating immune cells and the underlying chemokine and cytokine expression following selective ablation of Foxp3^+^ Treg in C57BL/6 mice was investigated.

## Methods

### Mice

BAC-transgenic DEREG mice have been described previously [20]. All animals were gender matched and control mice used in our experiments were non-transgenic littermates obtained from DEREG breeding. Mice were bred and maintained under specific pathogen-free condition in our animal facilities. All animals used in experiments were 8 weeks of age. The animal experiments were approved and authorized by the local authorities (Niedersächsisches Landesamt für Verbraucherschutz- und Lebensmittelsicherheit (LAVES), Oldenburg, Germany, permission number 33.12-42502-04-10/0150) and performed according to international guidelines on the use of laboratory animals [21].

### DT treatment

DEREG and WT control mice received 1 μg of diphtheria toxin (DT) intraperitoneally (ip) (Calbiochem, San Diego, CA, USA) in 100 μl PBS on days −2 and −1 corresponding to the infection.

### Intracerebral injection

Eight-week-old male DEREG mice and non-transgenic littermate controls were inoculated into the right hemisphere with 1.63 × 10^6^ plaque-forming units/mouse of the BeAn strain of TMEV in 20 μl DMEM (PAA Laboratories, Cölbe, Germany) with 2% fetal calf serum and 50 μg/kg gentamicin. Inoculation was carried out under general anesthesia with medetomidine (0.5 mg/kg, Domitor, Pfizer, Karlsruhe, Germany) and ketamine (100 mg/kg, ketamine 10%, WDT eG, Garbsen, Germany).

### Isolation of cells from brain

Mice were transcardially perfused with PBS and the brains were carefully dissected and collected in Hank’s balanced salt solution containing 15 mM HEPES and 5% glucose. The brain was cut into small pieces and digestion was carried out by incubating the tissue fragments in DMEM containing 0.5 mg/ml collagenase D (Roche, Mannheim, Germany) and 10 μg/ml DNase I (Roche, Mannheim, Germany) in an orbital shaker at 80 rpm for 30 minutes at 37°C. Tissue suspensions were then passed through a 70-μm mesh, pelleted, resuspended in a 40% isotonic Percoll solution (GE Healthcare, Uppsala, Sweden), and layered over 70% isotonic Percoll solution. After centrifugation at 2,100 rpm for 30 minutes at room temperature, the upper myelin layer was aspirated and the cells at the interface were collected, washed once with PBS and then used for further analyses.

### Antibodies and reagents

Fluorochrome conjugated antibodies specific for mouse, anti-CD3 (clone: 145-2C11), anti-CD4 (clone: RM4-5), anti-Foxp3 (clone: MF-14), anti-IFNγ (clone: XMG1.2) and anti-IL-17A (clone: TC11-18H10.1) were all purchased from Biolegend, San Diego, CA, USA. Anti-CD8 (clone: 53–6.7) was purchased from eBioscience, Frankfurt, Germany.

#### Flow cytometry

For cell surface staining, cells were preincubated with CD16/32 Fc-block antibody (clone: 93, eBioscience, Frankfurt, Germany) at room temperature (RT) for 10 minutes, and subsequently incubated with the indicated conjugated antibody cocktail for 30 minutes in a total volume of 100 μl of PBS containing 1% FBS. For live/dead staining, cells were incubated in fixable viability dye eFluor® 506 (1:1,000 dilution; eBioscience, Frankfurt, Germany) at 4°C for 30 minutes. For intracellular cytokine staining, cells were restimulated for 6 hours in complete DMEM (Gibco, Darmstard, Germany) in the presence of 50 ng/ml phorbol 12-myristate 13-acetate and 500 ng/ml ionomycin (both from Sigma-Aldrich, St. Louis, MO, USA). For the last 4 hours, brefeldin A (10 μg/ml; Sigma-Aldrich, St. Louis, MO, USA) was added to the cultures. Intracellular staining/transcription factor was performed using Foxp3 staining buffer set (eBioscience, Frankfurt, Germany) according to the manufacturers’ recommendations. Samples were acquired on LSR II (BD Biosciences, San Jose, CA, USA) and analyzed with FlowJo software (Tree Star, Ashland, OR, USA).

### Histology, immunohistochemistry and immunofluorescence

For histology and immunohistochemistry the cerebrum was fixed in 10% formalin for 24 hours, embedded in paraffin wax and stained with H & E. Inflammatory responses were graded based upon the degree of perivascular infiltrates (PVI) using a semiquantitative scoring system: 1 = 1 to 25 cells (mild); 2 = 26 to 50 cells (moderate); 3 = >50 cells per high power field (severe) as previously described [22]. Immunohistochemistry was performed using a polyclonal rabbit anti-CD3 antibody (DakoCytomation, Hamburg, Germany) for the detection of T cells. For blocking of the endogenous peroxidase, formalin-fixed, paraffin-embedded tissue sections were treated with 0.5% H_2_O_2_ diluted in methanol for 30 minutes at RT. Antigen retrieval was done by heating the sections in 10 mM Na-citrate buffer pH 6.0 for 20 minutes in a microwave oven (800 W). Subsequently, slides were incubated with the respective primary antibody overnight at 4°C. Biotinylated goat-anti-rabbit IgG diluted 1:200 (Vector Laboratories, Burlingame, CA, USA) was used as a secondary antibody. Sections used as negative controls for CD3 immunohistochemistry were incubated with rabbit normal serum at a dilution of 1:2,000 (Sigma-Aldrich Chemie GmbH, Taufkirchen, Germany). Slides were subsequently incubated with the peroxidase-conjugated avidin-biotin complex (ABC method, Vector Laboratories, Burlingame, CA, USA) for 30 minutes at RT. After the positive antigen-antibody reaction visualization by incubation with 3.3-diaminobenzidine-tetrachloride (DAB) in 0.1 M imidazole, sections were counterstained with Mayer’s hematoxylin.

For immunofluorescence staining, antigen retrieval was performed as mentioned above, following which the sections were blocked with 10% normal donkey serum. Slides were incubated with primary anti-IL10 (clone: M-18: Santa Cruz Biotechnology, Santa Cruz, CA, USA) along with anti-CD3, anti-glial fibrillary acidic protein (GFAP) (DakoCytomation, Hamburg, Germany) or anti-Iba1 (Wako, Osaka, Japan) antibodies for 2 hours at RT and after thorough washing the slides were incubated with fluorochrome conjugated secondary donkey anti-goat antibodies for 1 hour.

The density of positive cells/mm^2^ in the cerebral neuroparenchyma was manually counted in randomly selected cerebral areas with a 40-fold objective and a 10-fold calibrated eyepiece reticule (Olympus Europe, Hamburg, Germany).

#### RNA isolation

RNA was isolated from 10 to 40 mg tissue of each frozen brain using an Omni’s PCR Tissue Homogenizing Kit (Süd-Laborbedarf GmbH, Gauting, Germany) and RNeasy® Lipid Tissue Mini Kit (#74804; Qiagen GmbH, Hilden, Germany).

### Reverse transcription-polymerase chain reaction (RT-PCR)

Equal amounts of RNA were subsequently transcribed into cDNA with the High Capacity cDNA Reverse Transcription Kit with RNase Inhibitor (#4374966; Applied Biosystems®; Life Technologies GmbH, Darmstadt, Germany). RT-PCR for the quantification of TMEV (forward primer: 5′-GACTAATCAGAGGAACGTCAGC-3′; reverse primer: 5′-GTGAAGAGCGGCAAGTGAGA-3′) and three housekeeping genes: glyceraldehyde 3-phosphate dehydrogenase (GAPDH; forward primer: 5′-GAGGCCGGTGCTGAGTATGT-3′; reverse primer: 5′-GGTGGCAGTGATGGCATGGA-3′), β-actin (forward primer: 5′-GGCTACAGCTTCACCACCAC-3′; reverse primer: 5′-ATGCCACAGGATTCCATACC-3′), hypoxanthine-guanine phosphoribosyltransferase (HPRT; forward primer: 5′-GGACCTCTCGAAGTGTTGGA-3′; reverse primer: 5′-TTGCGCTCATCTTAGGCTTT-3′) in brain tissues was performed using the Mx3005P™ Multiplex Quantitative PCR System (Agilent Technologies Deutschland GmbH, Böblingen, Germany) and SYBR® Green I as DNA-binding dye as described [23,24]. Ten-fold serial dilution standards ranging from 10^8^ to 10^2^ copies/μl were used to quantify the results. A normalization factor achieved from the three housekeeping genes was calculated using the geNorm software version 3.4 to correct for experimental variations [25]. Specificity of each reaction was controlled by melting curve analysis.

For other gene expression analysis, quantitative real-time PCR was performed using the StepOne™ Real-Time PCR System and appropriate TaqMan probes (Applied Biosystems, Life Technologies GmbH, Darmstadt, Germany, Additional file 1: Table S1). The ΔΔCt method was applied to determine differences in the expression of *CCL2*, *CXCL10*, *interleukin*-*6* (*IL-6*), *interleukin-10* (*IL-10*), *inferferon-γ* (*IFNγ*), *tumor necrosis factor-α* (*TNF-α*) and *inducible NO synthase* (*NOS2*) *and granulocyte-macrophage colony stimulating factor* (GM-*CSF*) between uninfected and TMEV-infected animals that were previously treated with PBS or DT. Changes in mRNA expression levels were calculated after normalization to the arithmetic mean of *HPRT* and *GAPDH*.

### Statistical analysis

All statistical analyses were conducted using GraphPad Prism 5.0 (GraphPad Software, La Jolla, CA, USA). All data are expressed as group mean ± SD unless otherwise stated. The data generated from several mice from each group was analyzed using Student’s *t*-test. Results were considered statistically significant at *P* <0.05.

## Results

### Treg depletion prior to TMEV infection enhances the infiltration of T cells into the brain

**Figure 1:**
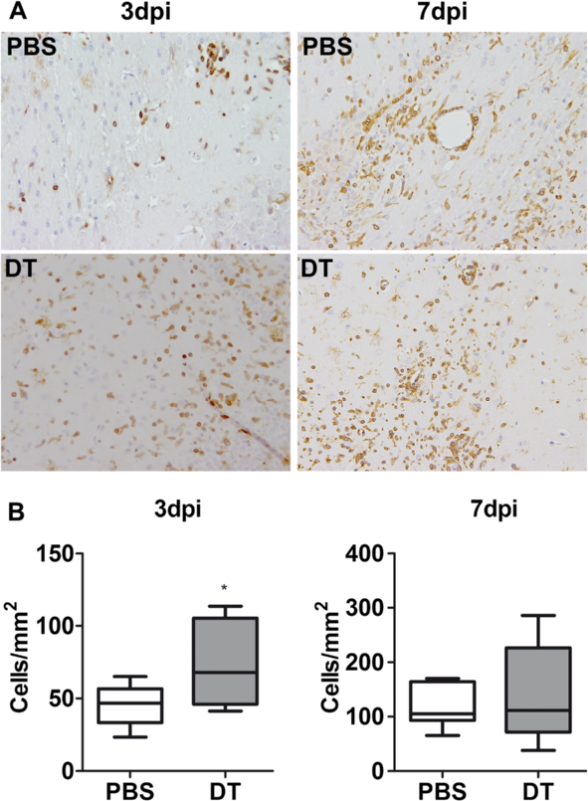
**Early recruitment of T cells into Theiler’s murine encephalomyelitis virus (TMEV)-infected brain in the absence of Tregs.** Following intraperitoneal administration of PBS or diphtheria toxin (DT), DEREG mice were intracerebrally infected with TMEV. **(A)** Immunohistochemistry of TMEV infected brains at 3 days post inoculation (dpi) (left panel) and 7 dpi (right panel) reveals higher numbers of CD3^+^ T cells only at 3 dpi in DT-treated mice (lower panel) compared to PBS-treated mice (upper panel). **(B)** Quantification of CD3^+^ T cells in the cerebral neuroparenchyma of 6 to 8 infected mice reveals a significantly increased number on T cells in DT-treated mice at 3 dpi. Box and whisker plots display median and quartiles with maximum and minimum values. *P-value <0.05.

In our initial attempts we assessed the outcome of Treg depletion on the acute response to TMEV infection. For this we employed the strategy of depleting Foxp3^+^ Treg prior to TMEV infection by ip administration of DT into DEREG mice, which express the DT receptor under the control of the *foxp3* locus [20]. Analysis of blood 1 day after DT injection revealed that most of the Foxp3^+^ CD4^+^ T cells were depleted from circulation (Additional file 2: Figure S1). Following intracerebral inoculation of the BeAn strain of TMEV into Treg-depleted and non-depleted mice, the inflammatory response and virus load were assessed. Histology revealed no differences in the degree of neuroinflammation between Treg-depleted and non-depleted mice (data not shown). Interestingly, despite the similar severity of encephalitis among the groups, we observed in immunohistochemistry a significantly higher number of CD3^+^ T cells in the cerebrum of Treg-depleted mice at 3 days post inoculation (dpi) “However, this difference in the number of T cells was transient and was not observed at 7 dpi (Figure 1A and B). Such differences were not evident among PBS- and DT-treated wild type (WT) littermate controls at these time points, thus confirming that transient increase in the number of T cells was the result of Treg depletion (Additional file 3: Figure S2A). These results suggest that Tregs might regulate the early recruitment of effector T cells to the sites of infection.

#### Predominantly IFN*γ-*producing T cells infiltrate into the brain

**Figure 2:**
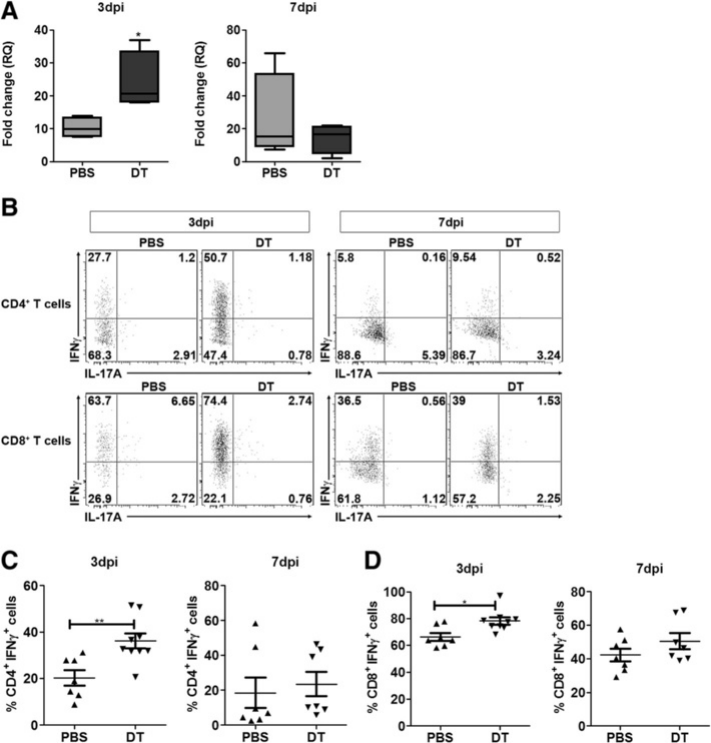
**Increased frequency of IFNγ-producing T cells found in the brain tissue of Treg-depleted mice post Theiler’s murine encephalomyelitis virus (TMEV) infection** ***.*** **(A)** RT-PCR of mRNA isolated from the brain of TMEV-infected DEREG mice treated with PBS or diphtheria toxin (DT) show an approximately 2-fold higher expression of IFNγ in Treg-depleted (DT) mice compared to non-depleted mice (PBS) at 3 days post inoculation (dpi). However, no significant difference can be observed at 7 dpi. The graph represents mean ± SD of the data obtained from four infected mice from each group. **(B)** Flow cytometry analysis of cells isolated from infected brains show a higher frequency of IFNγ-producing T cells CD4^+^ (upper panel) and CD8^+^ (lower panel) T cells at 3 dpi (left panel) and not at 7 dpi (right panel). No IL-17-producing cells were found in any of the groups. Data obtained by analyzing the CD3^+^CD4^+^ **(C)** and CD3^+^CD8^+^ cells **(D)** from 7 to 9 infected mice from each group is shown in this plot. *P-value <0.05, **P-value <0.01.

Our next attempt was to assess the effect of Treg depletion on the antiviral immune response in the CNS. IFNγ is a prominent effector molecule in antiviral immune defense and produced mainly by effector lymphocyte populations. Measuring the gene expression in infected brains by RT-PCR, we found, at 3 dpi, a 2-fold higher expression of IFNγ in Treg-depleted mice compared to non-depleted mice. No such differences in the expression of IFNγ were observed at 7 dpi between the two groups (Figure 2A). This coincides with the above mentioned observation where we found a transient increase of T cells at 3 dpi in the absence of Tregs. Therefore, we sought to isolate immune cells from the brain of infected mice and analyze the T cell subpopulations in terms of their effector cytokine secretion profile. At 3 dpi, only few T cells were detected among the cells harvested from the brain of mice with an unaltered Treg compartment. Upon Treg depletion prior to infection there was an increased infiltration of T cells as we observed an increased frequency of CD4^+^ and CD8^+^ T cells in the brain (Additional file 4: Figure S3). Further supporting our RT-PCR data, flow cytometric analysis revealed a significantly higher percentage of IFNγ-producing Th1 and CD8^+^ T cells among the cells isolated from the brain of Treg-depleted mice at 3 dpi but not at 7 dpi (Figure 2B and C). No IL-17-producing CD4^+^ Th17 cells were found at both time points analyzed, regardless of the Treg status (Figure 2B). It is noteworthy that frequencies of IFNγ-producing T cells in the spleen of Treg-depleted mice were significantly higher at 3 dpi and only marginally higher at 7 dpi compared to non-depleted mice (Additional file 5: Figure S4). Further, we could confirm that the observed effects were only due to Treg depletion in DEREG mice, as no such differences were observed in DT-treated WT littermate controls (Additional file 3: Figure S2B-E). Taken together, these results suggest that a strong IFNγ-producing T cell response is primed in the periphery in the absence of Tregs and this might lead to an early recruitment of these effector T cells into the brain.

### Depletion of Tregs prior to infection does not influence viral clearance in DEREG mice

**Figure 3:**
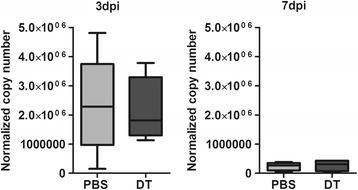
**Treg depletion has no impact on viral clearance.** Viral load in the infected brain was determined by quantification of Theiler’s murine encephalomyelitis virus (TMEV) RNA by RT-PCR. No significant differences in viral RNA copy numbers between Treg-depleted (diphtheria toxin; DT) or non-depleted mice (PBS) was observed either at 3 days post inoculation (dpi) or 7 dpi. In either case, the virus was efficiently eliminated as demonstrated by significantly lower number of viral RNA transcripts at 7 dpi. Five mice from each group were analyzed. Box and whisker plots display median and quartiles with maximum and minimum values.

Since we observed a higher frequency of IFNγ-producing CD4^+^ and CD8^+^ T cells infiltrating into the brain of TMEV-infected mice in the absence of Tregs at 3 dpi, we expected an enhanced and faster viral clearance in Treg-depleted mice. Therefore, we sought to assess the viral load by RT-PCR in the brain at 3 dpi and 7 dpi. Significantly lower copy numbers of viral RNA were detected at 7 dpi compared to 3 dpi, indicating a potent antiviral response in TMEV-infected mice. However, it appears that Treg depletion had no impact on the antiviral response as we did not observe any significant differences in the viral load between Treg-depleted and non-depleted mice neither at 3 dpi nor at 7 dpi (Figure 3).

### Treg depletion did not alter the expression of inflammatory mediators in the brain

**Figure 4:**
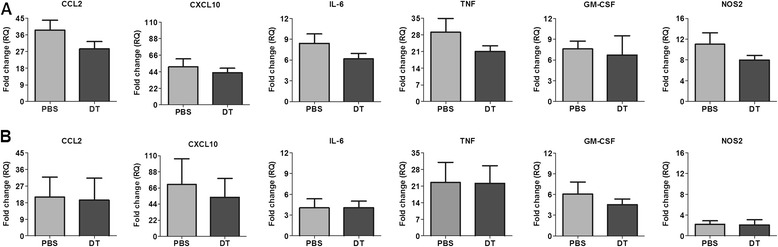
**Expression of pro-inflammatory mediators is unaffected by Treg depletion.** To study the local inflammatory response the gene expression of selected pro-inflammatory mediators (CCL2, CXCL10, GM-CSF, TNF-α, IL-6 and NOS2) was compared between Treg-depleted (diphtheria toxin; DT) and non-depleted (PBS) TMEV-infected mice at 3 days post inoculation (dpi) **(A)** and 7 dpi **(B)** by RT-PCR. Fold changes (RQ) was calculated by using ΔΔCt method (normalizing the ΔCt values obtained from infected mice to that of uninfected controls). The bar graphs represent mean ± SD of fold change in gene expression obtained by analyzing four mice from each group.

It was rather puzzling to have an increased frequency of IFNγ^+^ T cells and yet not to observe any difference in the viral load or tissue damage. Therefore, we studied the local inflammatory response triggered by TMEV infection in the absence of Tregs. We compared gene expression of certain inflammatory mediators in response to TMEV infection in the brain of Treg-depleted and non-depleted mice. Chemokines CCL2 and CXCL10, believed to be produced by resident glial cells and known to play a major role in the recruitment of monocytes and T cells from the periphery, were strongly induced upon TMEV infection (Figure 4A and B). Nevertheless, depletion of Tregs had no influence on their expression both at 3 dpi and 7 dpi. Similarly, a potent pro-inflammatory response was induced upon TMEV infection characterized by significantly increased expression of GM-CSF, IL-6, TNF-α, and NOS2 in these mice compared to uninfected controls. However, we did not detect any significant difference in the transcription of these genes between Treg-depleted and non-depleted mice (Figure 4A and B).

### IL-10 produced by microglia/macrophage compensates for the lack of Tregs

**Figure 5:**
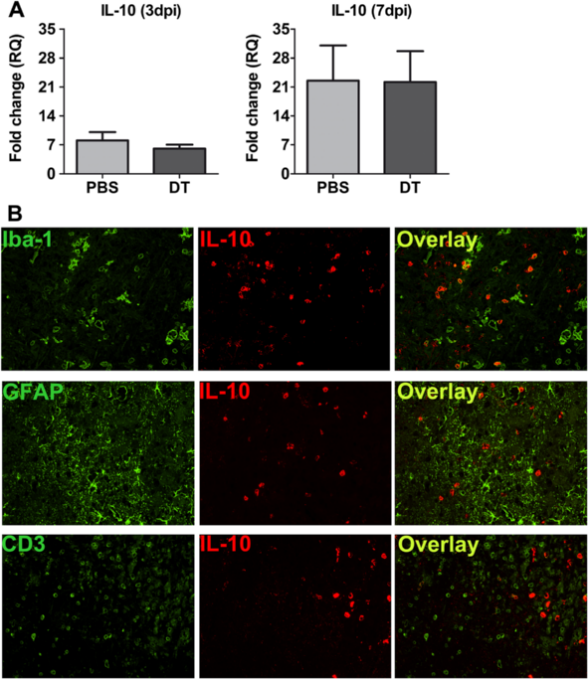
**Locally produced IL-10 might be responsible for anti-inflammatory response** ***.*** **(A)** *IL-10* gene expression at 3 days post inoculation (dpi) and 7 dpi in Treg-depleted (diphtheria toxin; DT) and non-depleted (PBS) mice was assessed by RT-PCR. The bar graphs represent mean ± SD of fold change in gene expression obtained by analyzing four mice from each group. **(B)** Double-immunofluorescence stainings of coronal brain sections using antibodies against IL-10 (red) in combination with Iba-1 (microglia), glial fibrillary acidic protein (GFAP) (astrocytes) or CD3^+^ (T cells) at 7 dpi in Treg-depleted mice show that microglia are the major source of IL-10.

Another interesting observation from the RT-PCR analysis was that the expression of the anti-inflammatory cytokine IL-10 in the brain of infected mice was unaffected despite of Treg-depletion. The amount of IL-10 expressed was similar in Treg-depleted and non-depleted mice both at 3 dpi and 7 dpi (Figure 5A). However, expression of IL-10 was increased by few folds at 7 dpi, suggesting a role of locally produced IL-10 in neuroprotection. In an attempt to delineate the source of IL-10 we performed immunofluorescence co-stainings of 7 dpi brain sections [18]. We found that the majority of IL-10 signals co-localized with Iba-1^+^ cells, indicating that microglia or infiltrating macrophages could be the source of IL-10 (Figure 5B). At the same time IL-10 was found lacking among the majority of CD3^+^ T cells and GFAP^+^ astrocytes. However, we failed to detect any significant difference in the number of Iba1^+^ IL-10^+^ cells between PBS- and DT-treated groups at this time point (data not shown). Taken together these results suggest that IL-10 produced by microglia/macrophages compensates for the lack of Tregs and might limit the extent of tissue damage resulting from increased infiltration of effector T cells.

## Discussion

In this study, we investigated the role of Foxp3^+^ Tregs during an acute viral infection in the CNS using DEREG mice in which Tregs can be selectively depleted by injecting DT. Our findings support the hypothesis that Tregs have no major influence in regulating immune responses against TMEV in the CNS of resistant C57BL/6 mice. However, this does not reflect an altogether null effect of Treg depletion as we did observe a transient increase in the number of effector Th1 and CD8^+^ T cells in the brain at an early stage of infection.

TME is a good model to study the pathogenesis of virus-mediated demyelinating diseases. In response to TMEV infection, CD4^+^ and CD8^+^ T cells are primed in the periphery, which subsequently infiltrate into the CNS and mount antiviral immune responses, leading to effective viral clearance within the first weeks of infection in resistant C57BL/6 mice. On the contrary, susceptible SJL mice show an inadequate viral clearance, which causes virus persistence and chronic demyelination [26,27]. One of several factors contributing to this phenomenon in SJL mice is the suppressive activity of infiltrating Tregs in the CNS together with insufficient effector CD8^+^ T cell responses [11,28]. It has been demonstrated that functional inactivation of Tregs prior to TMEV infection enhances viral clearance by augmenting CD8^+^ T cells, Th1 cells and antibody responses leading to delayed disease onset and progression [11]. These results suggest that Tregs lead to TMEV persistence by suppressing antiviral responses in mice with a susceptible background. On the other side, while inhibiting effector T cell function, Tregs eventually limit CNS inflammation and tissue damage. It has been shown by a previous study that Treg depletion prior to EAE induction increases disease severity [29]. Therefore, we intended to study the role of Tregs in resistant C57BL/6 mice, which mount an efficient TMEV-specific immunity. Interestingly, at 3 dpi we observed a significantly higher frequency of IFNγ-producing CD4^+^ and CD8^+^ T cells in the brain in the absence of Tregs. In contrast to findings in SJL mice, we did not observe any differences in the viral load between Treg-depleted and non-depleted mice suggesting that the anti-TMEV response in C57BL/6 mice is unaffected by the presence or absence of Tregs. This is in line with a recent report that used an alternative approach of adoptive transfer of *in vitro*-induced Tregs (iTreg) and found that early iTreg treatment exacerbated TMEV-induced demyelination in SJL mice while C57BL/6 mice were largely unaffected by such treatments [19]. In C57BL/6 mice, TMEV infection results in rapid expansion of effector CD4^+^ and CD8^+^ T cells, which are efficient in clearing the virus [26]. Therefore, it might not be surprising that a mere increase in T cell numbers would lead to significant differences in viral load. It should be noted that the CNS has a limited regenerative capacity and hence an unwarranted inflammation triggered by immune cells infiltrating from the periphery might result in CNS tissue damage. Early studies have shown that an increase in pro-inflammatory Th1 responses enhances the development of demyelinating disease in resistant as well as in susceptible mice following TMEV infection [30,31]. Hence, we expected that a heightened IFNγ response in the brain would trigger a stronger inflammatory response and cause more severe tissue damage. However, we neither observed early clinical signs in any of the groups nor any differences in the degree of tissue damage between Treg-depleted and non-depleted mice. This, we believe, is due to the fact that the inflammatory response in the brain is largely unaltered despite Treg depletion. Accordingly, the pro-inflammatory factors TNF-α, GM-CSF, IL-6 and NOS2 displayed similar expression levels in mice with unaltered or reduced amounts of Tregs in response to TMEV. Another striking observation was that the expression of the anti-inflammatory cytokine IL-10 was unaffected despite Treg depletion. This is interesting considering the fact that Tregs are an important source of IL-10 during CNS inflammation [29]. A recent study demonstrated that the expression of IL-10 during TMEV infection is mostly restricted to T cells; however, a portion of macrophages/microglia also contributes to IL-10 expression in the brain of acutely TMEV-infected mice [18]. We observed expression of IL-10 as early as at 3 dpi and was further increased at 7 dpi regardless of the presence or absence of Tregs. It is known that IL-10 has a dampening effect on inflammatory responses and plays a major role in inducing anti-inflammatory mechanisms in the brain [19]. In C57BL/6 mice, resident glial cells, especially M2-type microglia/macrophages, by producing IL-10 either in response to virus or due to heightened inflammatory response, might compensate for the lack of Tregs. Hence, we did not observe any damaging effects of additional effector T cells that infiltrate into the brain.

In conclusion, we could confirm that resistance to TMEV infection in C57BL/6 mice is largely due to the induction of effective CD4^+^ and CD8^+^ T cell responses, which is not influenced by Treg depletion. Furthermore, sustained expression of IL-10, mainly by resident glial cells during early infection, might limit the extent of damage caused by an unwanted immune response in the brain. While we believe that regulation by Tregs is strain-dependent, a significant role of local anti-inflammatory cytokines like IL-10 in neuroprotection during acute viral infections of the CNS cannot be ignored.

## Additional files

Additional file 1: Table S1. Taqman probes used for RT-PCR analysis. Additional file 2: Figure S1. Depletion efficiency in DEREG mice post DT treatment. Wild type (WT) and DEREG mice were treated ip with two doses of 1 μg/ml of DT or with PBS alone on consecutive days. One day later, percentage of CD4^+^ Foxp3^+^ cells in the blood was determined by flow cytometry. The majority of Tregs were depleted in DEREG mice treated with DT whereas Tregs in WT mice were unaffected. Six mice were analyzed from each group. ****P*-value <0.001. Additional file 3: Figure S2. Diphtheria toxin administration in to WT mice has no obvious effects. In parallel to DEREG infection, non-transgenic littermates (WT) treated with PBS and DT were infected with TMEV to test the off-target effects of DT. (A) Immunohistochemistry of coronal brain sections at 3 dpi (left panel) and 7 dpi (right panel) to detect CD3^+^ T cells showed no differences between PBS- and DT-treated mice. Box and whisker plots display median and quartiles with maximum and minimum values. (B-E) Flow cytometric analysis of cells isolated form the brain and spleen of WT mice at 3 dpi (B and C) and 7 dpi (D and E) to determine the frequency of IFNγ-producing CD4^+^ T cells (B and D) and CD8^+^ T cells (C and E). No significant differences were observed between PBS- and DT-treated mice. Each dot in the scatter plot represents the data from an individual mouse. Additional file 4: Figure S3. A transient increase in the frequency of CD4^+^ and CD8^+^ T cells post TMEV infection in Treg-depleted mice. Flow cytometric analysis of cells isolated from the brain of Treg-depleted (DT) and non-depleted (PBS) mice at 3 dpi and 7 dpi show higher frequency of CD4^+^ and CD8^+^ T cells among living cells at 3 dpi in Treg-depleted mice. ****P*-value <0.001. Additional file 5: Figure S4. Effector T cells were efficiently primed in the periphery in response to TMEV infection following Treg ablation. Percentage of IFNγ-producing CD4^+^ and CD8^+^ T cells in the spleen was compared between non-depleted and Treg-depleted mice at 3 dpi and 7 dpi. **P*-value <0.05, ****P*-value <0.001.

## Abbreviations

CNS: central nervous system
MS: multiple sclerosis
DT: diphtheria toxin
DPI: days post inoculation
PVI: perivascular infiltrates
IL: interleukin
NOS2: inducible nitric oxide synthase
IP: intraperitoneal
CXCL: C-X-C chemokine ligand
CCL: C-C chemokine ligand
IFN: interferon
TNF: tumor necrosis factor
GM-CSF: granulocyte macrophage colony stimulating factor
GAPDH: glyceraldehyde 3-phosphate dehydrogenase
HPRT: hypoxanthine-guanine phosphoribosyltransferase
GFAP: glial fibrillary acidic protein
TMEV: Theiler’s murine encephalomyelitis virus
TME: Theiler’s murine encephalomyelitis
RT-PCR: real-time polymerase chain reaction
RNA: ribonucleic acid
mRNA: messenger ribonucleic acid
cDNA: complementary deoxyribonucleic acid
