# Supplementary material for: Limited role of regulatory T cells during acute Theiler virus-induced encephalitis in resistant C57BL/6 mice
Source: J Neuroinflammation. 2014 Nov 13;11:180. doi: 10.1186/s12974-014-0180-9 (PMC4236492; doi:10.1186/s12974-014-0180-9)
Supplement: Additional file 1: Table S1. — Taqman probes used for RT-PCR analysis. [file 12974_2014_180_MOESM1_ESM.pdf]

Supplementary table 1: Taqman probes used for RT-PCR analysis

**Gene**	**Assay**
CCL2	Mm00441242_m1
CxCL10	Mm00445235_m1
IL-10	Mm00439616_m1
IL-6	Mm00446190_m1
CSF2	Mm00438328_m1
TNF-α	Mm00443258_m1
IFN-γ	Mm01168134_m1
NOS-2	Mm00440485_m1
HPRT	Mm00446968_m1
GAPDH	Mm99999915_g1
