# Supplementary figures and images for: Limited role of regulatory T cells during acute Theiler virus-induced encephalitis in resistant C57BL/6 mice
Source: J Neuroinflammation. 2014 Nov 13;11:180. doi: 10.1186/s12974-014-0180-9 (PMC4236492; doi:10.1186/s12974-014-0180-9)

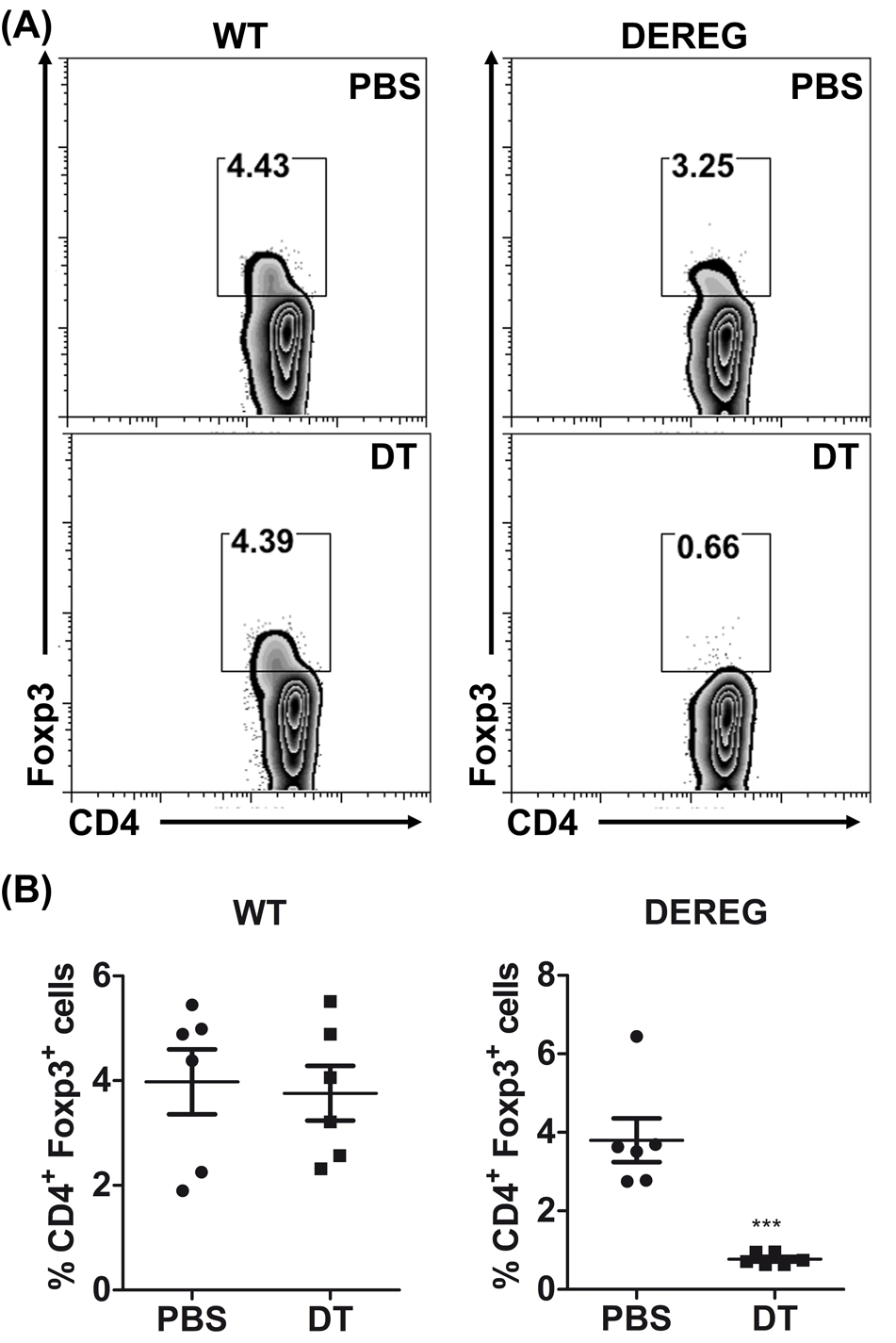

Supplement: Additional file 2: Figure S1. — Depletion efficiency in DEREG mice post DT treatment. Wild type (WT) and DEREG mice were treated ip with two doses of 1 μg/ml of DT or with PBS alone on consecutive days. One day later, percentage of CD4+ Foxp3+ cells in the blood was determined by flow cytometry. The majority of Tregs were depleted in DEREG mice treated with DT whereas Tregs in WT mice were unaffected. Six mice were analyzed from each group. ***P-value <0.001. [file 12974_2014_180_MOESM2_ESM.tif]

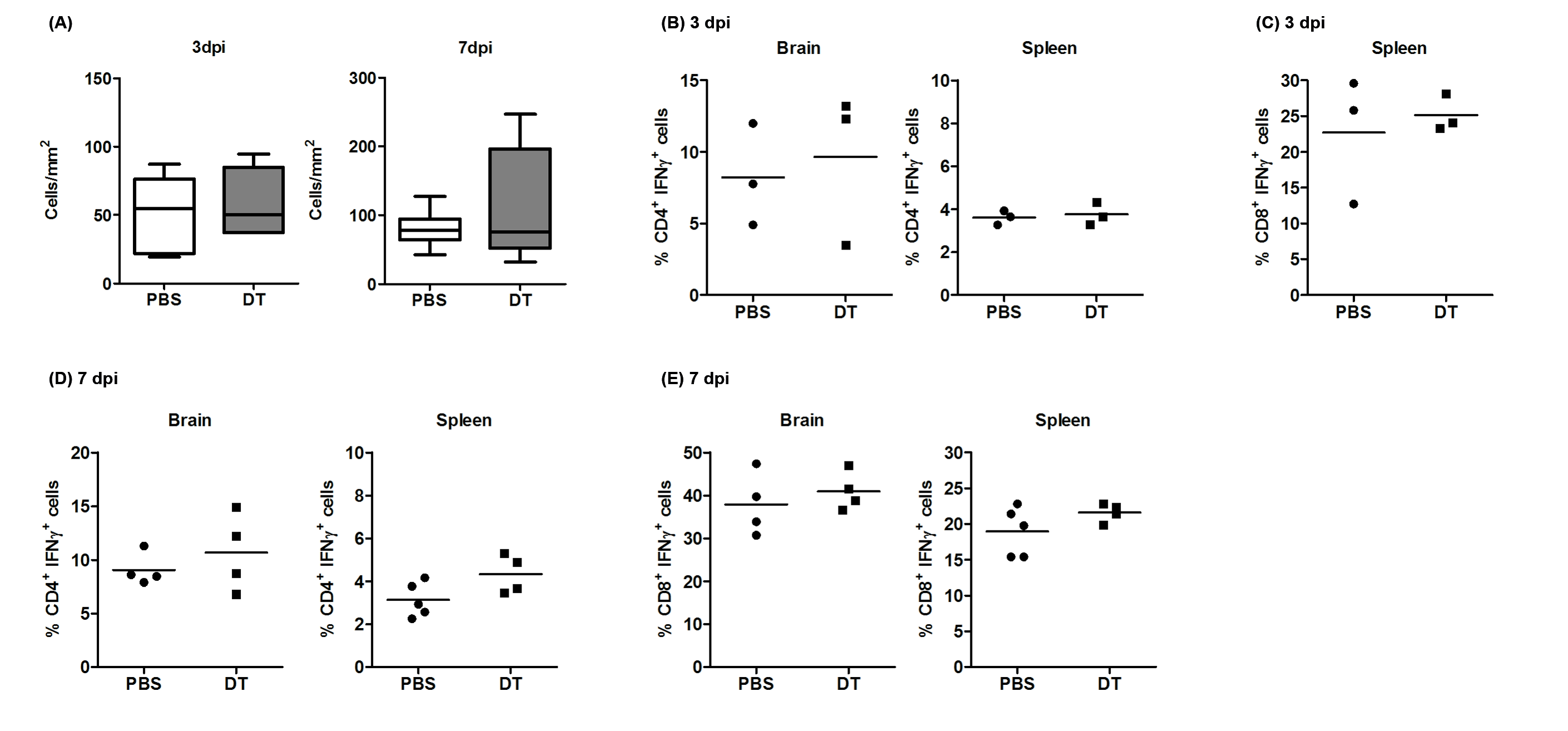

Supplement: Additional file 3: Figure S2. — Diphtheria toxin administration in to WT mice has no obvious effects. In parallel to DEREG infection, non-transgenic littermates (WT) treated with PBS and DT were infected with TMEV to test the off-target effects of DT. (A) Immunohistochemistry of coronal brain sections at 3 dpi (left panel) and 7 dpi (right panel) to detect CD3+ T cells showed no differences between PBS- and DT-treated mice. Box and whisker plots display median and quartiles with maximum and minimum values. (B-E) Flow cytometric analysis of cells isolated form the brain and spleen of WT mice at 3 dpi (B and C) and 7 dpi (D and E) to determine the frequency of IFNγ-producing CD4+ T cells (B and D) and CD8+ T cells (C and E). No significant differences were observed between PBS- and DT-treated mice. Each dot in the scatter plot represents the data from an individual mouse. [file 12974_2014_180_MOESM3_ESM.tif]

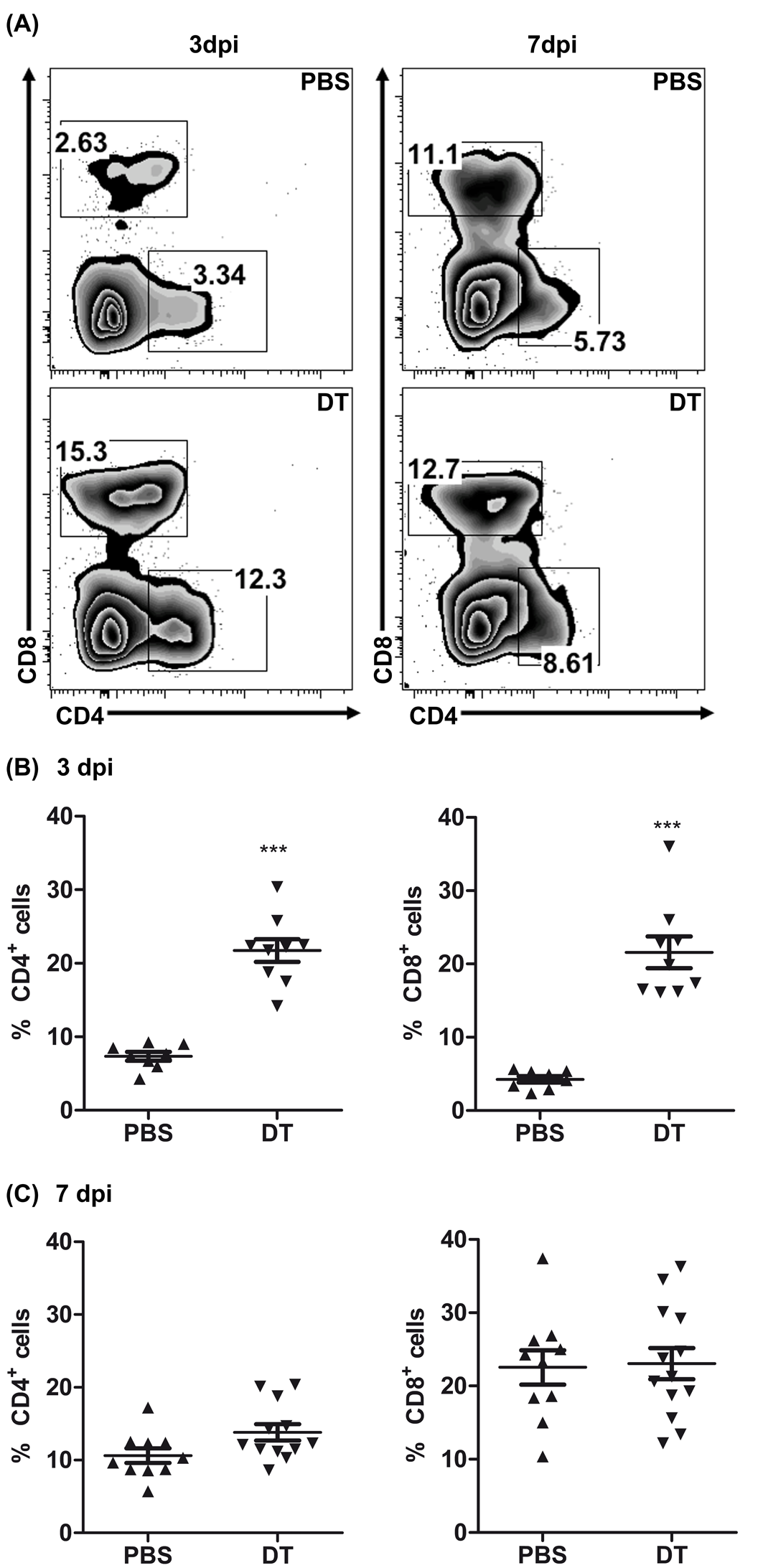

Supplement: Additional file 4: Figure S3. — A transient increase in the frequency of CD4+ and CD8+ T cells post TMEV infection in Treg-depleted mice. Flow cytometric analysis of cells isolated from the brain of Treg-depleted (DT) and non-depleted (PBS) mice at 3 dpi and 7 dpi show higher frequency of CD4+ and CD8+ T cells among living cells at 3 dpi in Treg-depleted mice. ***P-value <0.001. [file 12974_2014_180_MOESM4_ESM.tif]

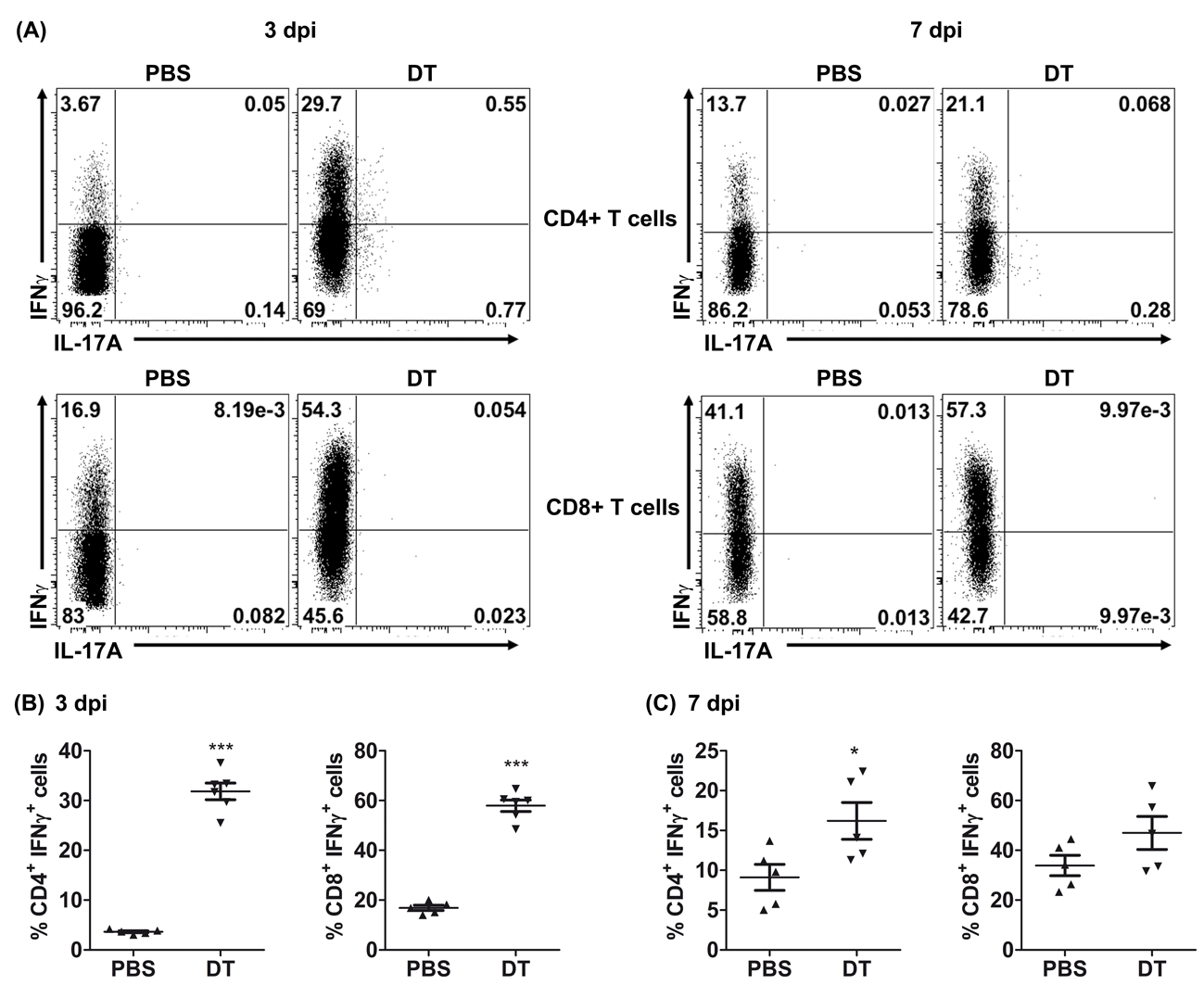

Supplement: Additional file 5: Figure S4. — Effector T cells were efficiently primed in the periphery in response to TMEV infection following Treg ablation. Percentage of IFNγ-producing CD4+ and CD8+ T cells in the spleen was compared between non-depleted and Treg-depleted mice at 3 dpi and 7 dpi. *P-value <0.05, ***P-value <0.001. [file 12974_2014_180_MOESM5_ESM.tif]
